# Inhibition of Survivin Reduces HIF-1α, TGF-β1 and TFE3 in Salivary Adenoid Cystic Carcinoma

**DOI:** 10.1371/journal.pone.0114051

**Published:** 2014-12-08

**Authors:** Yu-Fan Wang, Si-Rui Ma, Wei-Ming Wang, Cong-Fa Huang, Zhi-Li Zhao, Bing Liu, Wen-Feng Zhang, Yi-Fang Zhao, Lu Zhang, Zhi-Jun Sun

**Affiliations:** 1 The State Key Laboratory Breeding Base of Basic Science of Stomatology & Key Laboratory of Oral Biomedicine Ministry of Education, School and Hospital of Stomatology, Wuhan University, Wuhan, China; 2 Department of Oral Maxillofacial-Head Neck Oncology, School and Hospital of Stomatology, Wuhan University, Wuhan, China; National Health Research Institutes, Taiwan

## Abstract

In the present study, we explored the expression and correlation of survivin with HIF-1α, TGF-β1 and TFE3 in adenoid cystic carcinoma (AdCC). The expression of survivin, HIF-1α, TGF-β1 and TFE3 was assessed by immunohistochemical staining of a tissue microarray containing tissue samples of normal salivary gland (NSG), pleomorphic adenoma (PA) and AdCC. Correlation analysis of these proteins revealed that increased survivin expression was associated with the overexpression of HIF-1α (*P*<0.001, r = 0.5599), TGF-β1 (*P*<0.001, r = 0.6616) and TFE3 (*P*<0.001, r = 0.7747). The expression of survivin, HIF-1α, TGF-β1 and TFE3 was not correlated with the pathological type of human AdCC (*P*>0.05). Selective inhibition of survivin by YM155 and siRNA significantly reduced human SACC-83 cell proliferation, with the corresponding decrease in expression of HIF-1α, TGF-β1 and TFE3. The data indicate that the overexpression of survivin in AdCC is related to HIF-1α, TGF-β1 and TFE3. We hypothesize from these findings that the inhibition of survivin may be a novel strategy for neoadjuvant chemotherapeutic and radiosensitive treatment of AdCC.

## Introduction

Adenoid cystic carcinoma (AdCC) is one of the most common primary malignancies of the major and minor salivary glands [1]. Unlike head and neck squamous cell carcinoma, AdCC is characterized by neural infiltration and abundant angiogenesis [2]. Most patients with AdCC (80%–90%) die due to a high incidence of distant metastasis and recurrence [3]. Although several biomarkers contribute to this bleak scenario, the underlying molecular mechanisms of AdCC remain unknown. Therefore, the elucidation of the molecular events of AdCC is urgently required. Pathologically, AdCC is characterized by basaloid tumor consisting of epithelial and myoepithelial cells in variable morphologic configurations, including tubular, cribriform and solid patterns [4]. Some AdCC especially solid type may have difficult of differential diagnosis from mucoepidermoid carcinoma (MEC). Pleomorphic adenoma (PA) is and most common benign tumor of salivary gland with epithelial and modified myoepithlial elements intermingles with tissue of mucoid, myxoid or chondroid appearance [4].

Baculoviral inhibitor of apoptosis repeat-containing 5 (BIRC5), encodes a protein known as survivin that is a member of the inhibitor of apoptosis (IAP) family [5]. The role of survivin as a potential prognostic marker has been demonstrated extensively in various human cancers [6], [7]. Indeed, previous studies consistently confirm that a high expression of survivin is associated with poor prognosis and survival of AdCC [8], [9]. However, the mechanism underlying the effect of survivin on the clinicopathological characteristics and prognosis of AdCC remain unclear.

Of interest, hypoxia is a common environmental stress involved in cancer [10]. It has been demonstrated that intratumoral hypoxia can stimulate cell proliferation, angiogenesis, invasion and metastasis and is also responsible for treatment resistance in carcinoma[11]. Hypoxia-inducible factor-1 alpha (HIF-1α), one of the three homologues of the HIF alpha subunit (HIF-1α, HIF-2α, and HIF-3α), has been regarded as an important marker of tumor hypoxia [12], [13]. Under normoxia, HIF-1α is degraded, whereas during hypoxia, this process is inhibited. Stabilized HIF-1α is transported to the nuclear, where it heterodimerizes with HIF-1β to transactivate the expression of numerous hypoxia-responsive genes, such as VEGF and GLUT1 [14]. Interestingly, HIF-1α has been found to directly bind to the survivin promoter and up-regulate the transcriptional activity of survivin [15]. A positive correlation was observed in terms of increased HIF-1α and survivin expression in colon, pancreatic and breast cancer cell lines [16]. Transforming growth factor-beta 1 (TGF-β1) may coordinate with HIF-1α and regulates a diverse array of cellular and physiological processes such as migration, cell survival, fibrosis and epithelial-mesenchymal transition (EMT) [17]–[19]. Previous studies have shown that TGF-β1 regulates the expression of survivin in cancers [20]–[23]. Transcription factor E3 (TFE3), a member of the MiTF/TFE family of transcription factors, plays an important role in papillary renal carcinoma and melanoma [24], [25]. Moreover, it has been proved that TFE3 is a novel but important transcription factor in the canonical TGF-β1 signaling pathway [26]. So far, there is limited evidence to show the over expression of TFE3 in solid tumors other than papillary renal carcinoma and melanoma. These observations prompted us to examine the relationship among surviving, HIF-1α, TGF-β, and TGE3.

To date, no comprehensive analysis has been performed on the expression of survivin, HIF-1α, TGF-β1 and TFE3 in AdCC, and fewer studies have investigated the association between the expression of survivin and HIF-1α, TGF-β1 and TFE3. In this research, we explore the expression of these molecules in AdCC using a tissue microarray, investigate HIF-1α, TGF-β1 and TFE3 expression by direct inhibit suvivin and analyze the association between these factors.

## Materials and Methods

### Ethics statement, patient samples and tissue microarray (TMA) construction

This study was carried out in accordance with the ethical standards of the Helsinki Declaration and was approved by the Medical Ethics Committee of the Hospital of Stomatology, Wuhan University (PI: Zhi-Jun Sun). A written informed consent, to participate in the study, was obtained from each individual before surgery. A total of 74 surgically resected human adenoid cystic carcinoma, 25 normal salivary glands (NSGs), 12 pleomorphic adenoma (PA) and 8 mucoepidermoid carcinoma (MEC) specimens were obtained from the Department of Oral and Maxillofacial Surgery, School and Hospital of Stomatology Wuhan University. Histological classification was performed and classified according to the World Health Organization classification system [4]. A tissue microarray (T12-412-1) was constructed in collaboration with the Shanghai Biochip Company (Shanghai, China). Tissues were confirmed by 2 pathologists, and 1.5 mm punch tissue core were obtained from paraffin-embedded formalin-fixed tissue blocks from each patient. Two donor blocks from AdCC patients failed to obtain characteristic AdCC tissue in the block. Therefore, the final established block consist with a total of 117 validate cores of tissue samples, representative of 72 patients with adenoid cystic carcinoma (cribriform pattern: n = 28, tubular pattern: n = 24, and solid pattern: n = 20), 25 normal salivary glands (NSG), 12 pleomorphic adenoma (PA) and 8 patients with mucoepidermoid carcinoma (MEC) in the cohort. Serial sections of 3 µm-width from the resulting tissue microarray blocks were prepared using standard techniques.

### Cell culture, drugs, siRNA and cell immunofluorescence

The human adenoid cystic carcinoma cell line (SACC-83) was cultured in Dulbecco's Modified Eagle's Medium (DMEM) containing 10% fetal bovine serum (Gibco, Grand Island, NY, USA)[27]–[29]. YM155 (Selleck, TX, USA), a small molecule that inhibits survivin, was dissolved in DMEM to produce a stock solution (10 mmol/l) that was aliquoted and stored at −20°C [30]. BIRC5 siRNA and HiPerfect RNA interference were performed as previous described [31]. Briefly, SACC-83 cells were seeded in 6 cm culture dishes and allowed to grown to 80% confluence, transfected with BIRC5 siRNA or negative control siRNA (Qiagen, 5 µM final concentration) with Hiperfect transfection reagent (Qiagen) according to the manufacturer's instruction. The knock down efficiency with at least 90% decrease of BIRC5 siRNA protein at a indicated time (48 h) were confirmed by western blot as previous described [31]. Inhibition of survivin using small molecule YM155 (5 nM) were performed for 48 h. Cell immunofluroscence were performed as previous described [31] and detail described. Cells immunofluroscence were photographed and by confocal laser scanning microscopy (CLSM-310, Zeiss, Germany).

### Western Blot

SACC83 cells were treated with the indicated concentrations BIRC5 siRNA, negative control siRNA, YM155 in DMEM for 48 h. Then the cells were lysed in T-PER (Pierce, Rockford, IL), and the total protein was separated using 12% SDS-polyacrylamide gel electrophoresis and transferred onto polyvinylidene fluoride membranes (Millipore Corporation, Bil-lerica, MA). Then blots were developed by enhanced chemiluminescence detection kit (West Pico, Thermo). GAPDH was detected on the same membrane and used as a loading control as previous described [32].

### Immunohistochemistry, scoring system, Hierarchical clustering and data visualization

Immunohistochemistry was carried out as previously described [33]. The primary antibodies were against survivin (1∶200; Cell Signaling Technology, MA, USA), TGF-β1 (1∶200; Epitomics, CA, USA), HIF-1α (1∶500; Novus, CO, USA) and TFE3 (1∶400; Novus, CO, USA). TMA slides were incubated with primary antibodies overnight at 4°C. Then TMA was incubated with biotin-labeled secondary antibody, streptavidin peroxidase and visualized by incubation with 3, 3′-diaminobenzidine as previously described [33]. Slices were scanned using an Aperio ScanScope CS scanner (Vista, CA, USA) with background substration for each slice, and quantified using Aperio Quantification software (Version 9.1) for membrane, nuclear, or pixel quantification. An area of interest was selected either in the epithelial or the cancerous area for scanning and quantification. Histoscore of membrane and nuclear staining was calculated as a percentage of different positive cells using the formula (3+) ×3+ (2+) ×2+ (1+) ×1. Histoscore of pixel quantification was calculated as total intensity/total cell number [31]. The threshold for scanning of different positive cells was set according to the standard controls provided by Aperio [31], [34]. In Microsoft excel, the staining scores were converted into scaled values centered on zero, and expression data were recorded from −3 to +3 [33]. After the hierarchical analysis was achieved by Cluster 3.0 with average linkage based on Pearson's correlation coefficient, the results were visualized using Java TreeView 1.0.5 [35]. The clustered data were arranged with markers on the horizontal axis and tissue samples on the vertical axis. Two biomarkers with a close relationship were located next to each other.

### Statistical analysis

Statistical data analysis was performed with GraphPad Prism 5.03 (GraphPad Software, Inc., La Jolla, CA) statistical packages. Mann–Whitney *U* test or student *t* test was used to evaluate differences in the protein difference. One-way ANOVA followed by the post-Tukey or Bonferroni multiple comparison tests was used to analyze the differences in immunostaining. Two-tailed Pearson statistics were used for correlated expression of these markers after confirmation of the sample with Gaussian distribution. *P*<0.05 was considered significantly different.

## Results

### Hematoxylin and eosin stain of tissue arrays

**Figure 1:**
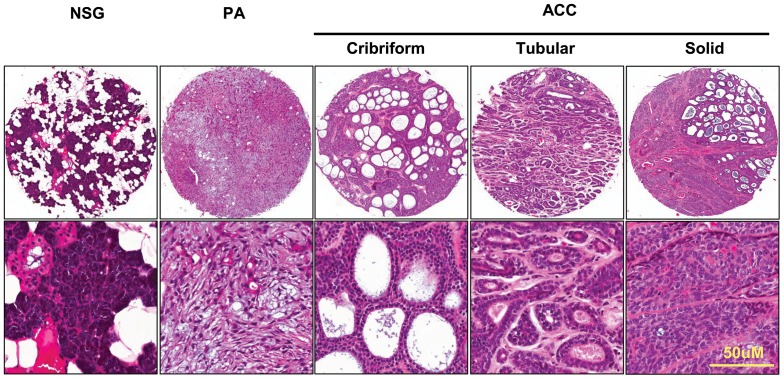
Image of tissue array. The HE staining of normal salivary gland (NSG), pleomorphic adenoma (PA) and cribriform, tubular, and solid adenoid cystic carcinoma (AdCC). Scale bars, 50 µm.

**Figure 2:**
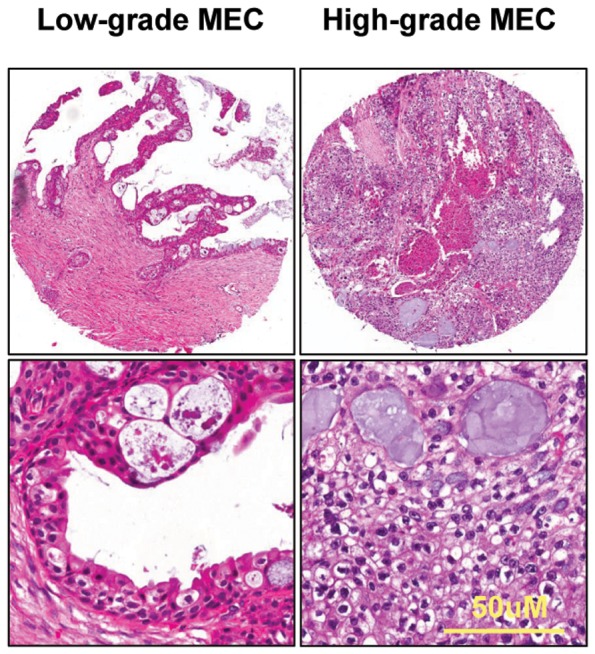
Image of tissue array. The HE staining of mucoepidermoid carcinoma (MEC, low grade and high grade). Scale bars, 50 µm.

The normal salivary gland and the tumor of the salivary gland array had a good morphology. HE-stained tissue arrays are highly representative of their donor tissues. We selected some representative pictures from the tissue array. The pictures displayed the typical pathological features of AdCC (Fig. 1) and MEC (Fig. 2).

### Expression of survivin, HIF-1α, TGF-β1 and TFE3 in the normal salivary gland (NSG), pleomorphic adenoma (PA) and AdCC

**Figure 3:**
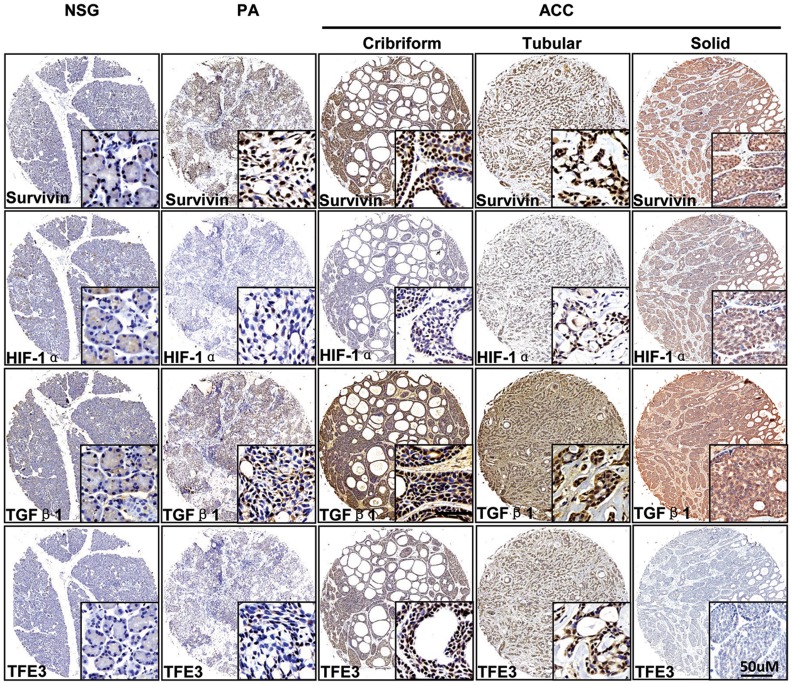
The expression of survivin, HIF-1α, TGF-β1 and TFE3 in the tissue samples of the NSG, PA and AdCC. Representative immunohistochemical staining (IHC) of survivin, HIF-1α, TGF-β1 and TFE3 in human AdCC tissue (right) as compared with normal salivary gland (NSG) and pleomorphic adenoma(PA, left). Scale bars, 50 µm.

**Figure 4:**
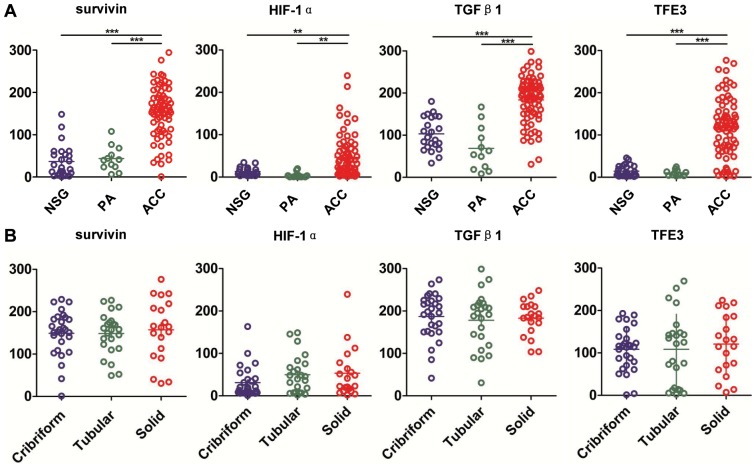
Quantatitive histoscores of survivin, HIF-1α, TGF-β1 and TFE3 expression in NSG, PA and AdCC. (A) Survivin, HIF-1α, TGF-β1 and TFE3 levels in AdCC were significant higher as compared with normal salivary gland (NSG) or pleomorphic adenoma (PA). (B) The expression of survivin, HIF-1α, TGF-β1 and TFE3 was not correlated with the pathological type of human AdCC. (Mean ± SEM, **, *P*<0.01, ***, *P*<0.001).

Normal salivary gland samples (18 parotid glands and 7 submandibular glands), 12 PA samples and 72 AdCC samples were selected for the immunohistochemical staining of survivin, HIF-1α, TGF-β1 and TFE3 antibody-stained array (S1 Datasheet). The protein expression of survivin, HIF-1α, TGF-β1 and TFE3 were consistent in all the tissues. Survivin staining was mostly located in the nuclear with very little cytoplasmic staining in the myoepithelial cell (Fig. 3). As shown in Fig. 4A, AdCC cells showed significant strong staining for survivin as compared with PA (*P*<0.001) and NSG (*P*<0.001). There was a slight increase in survivin staining in PA as compared with NSG, though the difference was not statistically significant (*P*>0.05). HIF-1α and TFE3 staining were located mostly in the nuclear of the AdCC tumor cells as compared with PA (Figs. 3 and 4A, *P*<0.01 for HIF-1α and *P*<0.001 for TFE3) and NSG (Figs. 3 and 4A, *P*<0.01 for HIF-1α and *P*<0.001 for TFE3). TGF-β1 was located mostly in the cytoplasm of the AdCC tumor cells (Fig. 3). TGF-β1 was highly expressed in AdCC tissues as compared with PA (Fig. 4A, *P*<0.001) and NSG (Fig. 3A, *P*<0.001). The protein expression of survivin, HIF-1α and TFE3 in AdCC was slightly higher in the solid type as compared with the cribriform and tubular type, though the difference between the 3 markers was not statistically significant (Fig. 4B).

### Expression of survivin, HIF-1α, TGF-β1 and TFE3 in mucoepidermoid carcinoma (MEC)

**Figure 5:**
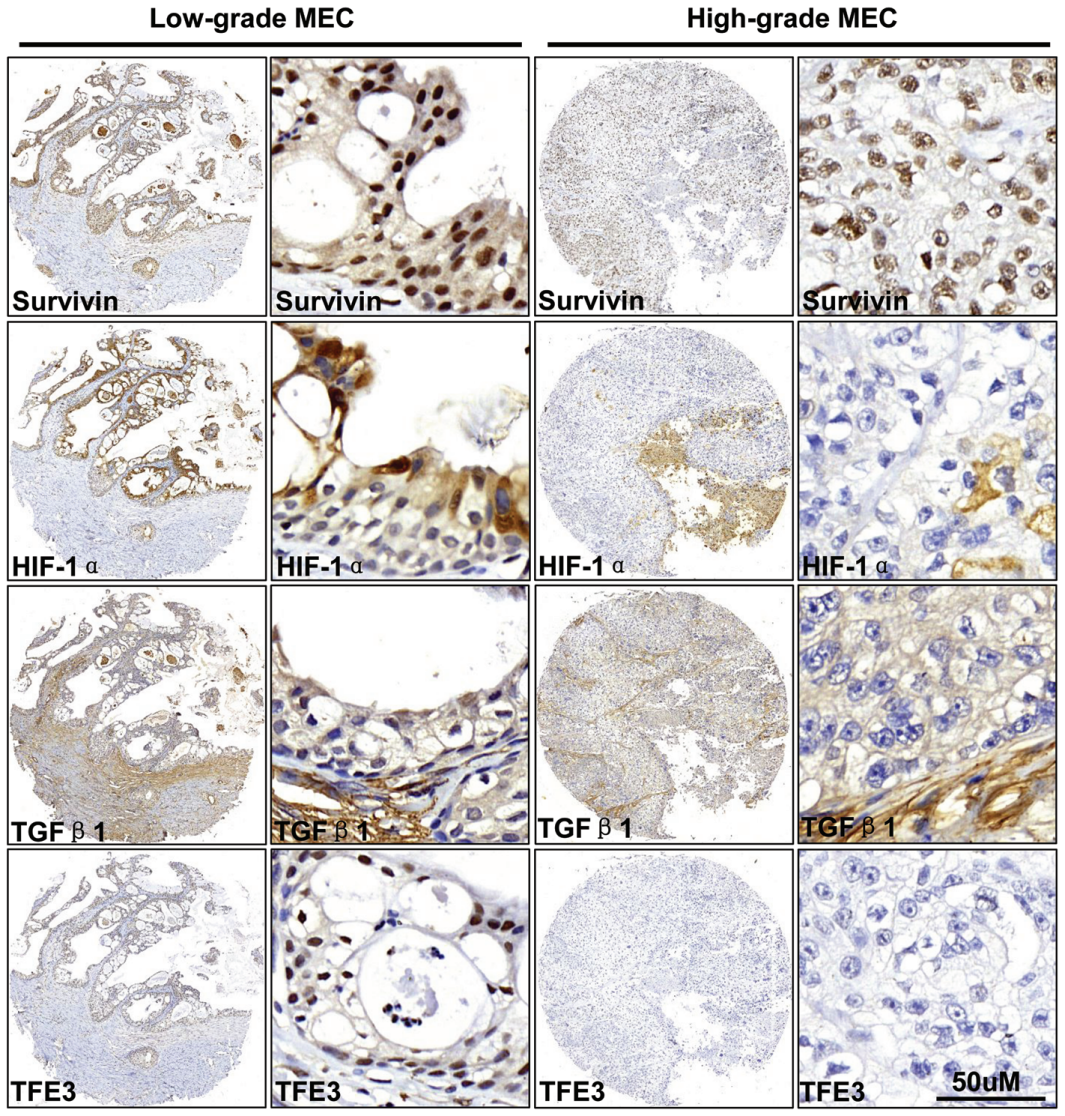
The expression of survivin, HIF-1α, TGF-β1 and TFE3 in mucoepidermoid carcinoma (MEC). Representative immunohistochemical staining (IHC) of survivin, HIF-1α, TGF-β1 and TFE3, in high grade MEC (right) and low grade MEC (left). Scale bars, 50 µm.

**Figure 6:**
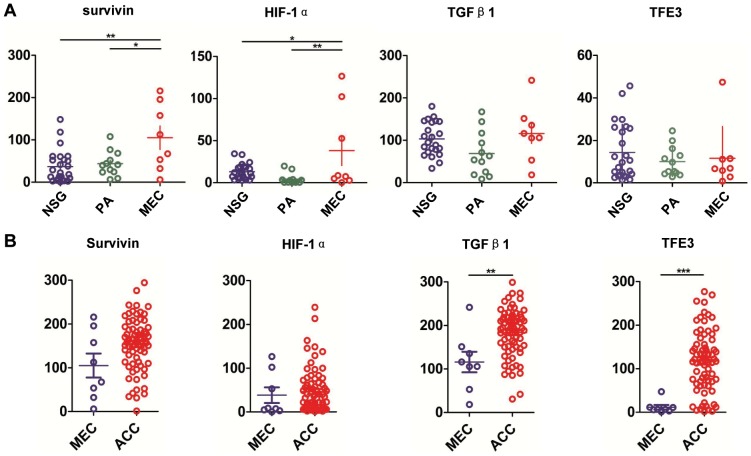
Quantatitive histoscores of survivin, HIF-1α, TGF-β1 and TFE3 expression in NSG, PA, AdCC and MEC. (A) Survivin and HIF-1α levels in mucoepidermoid carcinoma (MEC) were significantly higher than in normal salivary gland (NSG) and in pleomorphic adenoma (PA). The expression of TGF-β1 and TFE3 in MEC was not significantly different as compared with (NSG) or (PA). (B) TGF-β1 and TFE3 levels in AdCC were significantly higher than in MEC. The expression of survivin and HIF-1α in MEC were not significantly different as compared with AdCC. (Mean ± SEM, *, *P*<0.05,**, *P*<0.01, ***, *P*<0.001).

Pathologically, AdCC may have difficult to distinct from high grade MEC. To further explore the variable expression patterns of these molecules in AdCC and MEC, we examined the expression of survivin, HIF-1α, TGF-β1 and TFE3 in MEC. As shown in Fig. 5, the expression of survivin was observed in both mucus-producing cells and epidermoid cells. HIF-1α immunostaining was mainly in the cytoplasm of mucus-producing cells and less in the epidermoid cells. TGF-β1 expression was mainly in the stroma cells and was mildly expressed in the mucus-producing cells or epidermoid cells (Fig. 5). Nuclear stain of TFE3 was strong in low grade MEC with expression mainly in mucus-producing cells and was infrequent in epidermoid cells of high grade MEC (Fig. 5). The levels of survivin and HIF-1α in MEC were significantly higher than in NSG (*P*<0.01 for survivin and *P*<0.05 for HIF-1α, Figs. 5 and 6A) or PA (*P*<0.05 survivin and *P*<0.01 for HIF-1α, Figs. 5 and 6A). There is no difference in TGF-β1 and TFE3 expression in MEC compared to NSG or PA (*P*>0.05, Fig. 6A). In addition, there is no difference in survivin and HIF-1α expression in MEC compared to AdCC (*P*>0.05, Fig. 6B). Expression of TGF-β1 and TFE3 in AdCC was significantly increased in AdCC as compared with MEC (*P*<0.01 for TGF-β1 and *P*<0.001 for TFE3, Fig. 6B).

### The relationship between the expression of survivin, HIF-1α, TGF-β1 and TFE3 in AdCC

**Figure 7:**
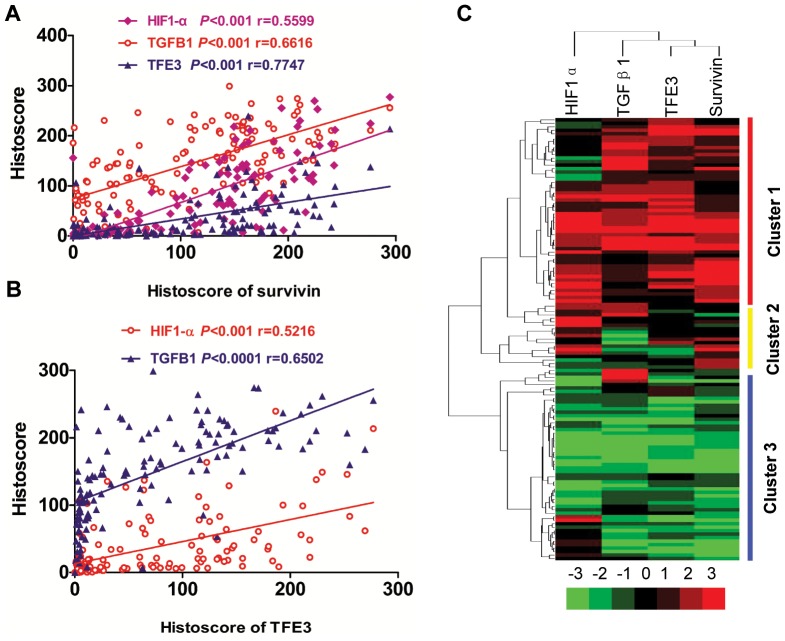
Correlation of survivin with HIF-1α, TGF-β1 and TFE3 in the human AdCC tissue array. (A) The expression of survivin had significant correlations with HIF-1α (*P*<0.001, r = 0.5599), TGF-β1 (*P*<0.001, r = 0.6616) and TFE3 (*P*<0.001, r = 0.7747) by using the Pearson correlation coefficient test in a human AdCC tissue array. (B) The expression of TFE3 is not only significantly related with HIF-1α (*P*<0.001, r = 0.5216) but also with TGF-β1 (*P*<0.0001, r = 0.6520) by using the Pearson correlation coefficient test in the human AdCC tissue array. (C) Hierarchical clustering of survivin with HIF-1α, TGF-β1 and TFE3 in the human AdCC tissue array. The histoscore based on quantification using the Aperio quantification software and statistics with Graph Pad Prism5. Mean ± SEM, 2-tailed Pearson correlation statistics.

To determine the biological effect and the relationship between survivin, HIF-1α, TGF-β1 and TFE3 in NSG, PA and AdCC, we evaluated the correlation of expression of survivin with the immunohistochemical protein expression of HIF-1α, TGF-β1 and TFE3 using the Aperio Scan Scope, a rather objective computer-based scanning and quantification technology. The Spearman rank correlation coefficient test and the linear tendency test were used. The statistical analyses demonstrated that the expression of higher levels of survivin was statistically associated with HIF-1α (*P*<0.001, r = 0.5599), TGF-β1 (*P*<0.001, r = 0.6616), and TFE3 (*P*<0.001, r = 0.7747) (Fig. 7A). This indicated an obvious positive correlation between survivin expression and HIF-1α, TGF-β1 and TFE3. In this study, we reported for the first time that TFE3 expression is highly expressed in AdCC with a similar expression pattern of HIF-1α, TGF-β1 and survivin. It is interesting to note that the high expression of TFE3 in AdCC is not only related with high expression of TGF-β1 (*P*<0.0001, r = 0.6502) but also correlated with HIF-1α (*P*<0.001, r = 0.5216) as shown in Fig. 7B. The data further demonstrated that high expression of survivin in AdCC is closely related to high expression of hypoxia related HIF-1α, TGF-β1 and TFE3. In the heat-map shown in Fig. 7C, the length and the subdivision of the branches indicated the correlation among the tested cases (left) as well as the markers (top). As expected, survivin, HIF-1α, TGF-β1 and TFE3 clustered together. TFE3 was clustered closer to survivin expression as compared with HIF-1α and TGF-β1. Of interest, using this clustering analysis, most of the AdCC cases (cluster 1 and 2) were distinct from PA and NSG (cluster 3), reflecting the significant differences in survivin, HIF-1α, TGF-β1 and TFE3 staining in AdCC.

### Inhibition of survivin by YM155 reduces HIF-1α, TGF-β1 and TFE3 expression in human SACC-83 cell line

**Figure 8:**
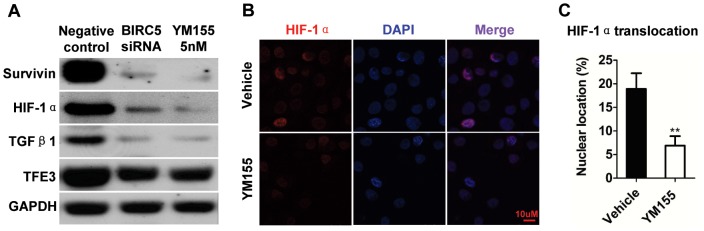
Inhibition of survivin decreased the expression of HIF-1α, TGF-β1 and TFE3 in human SACC-83 cell line. (A) The protein expression of survivin, HIF-1α, TGF-β1 and TFE3 with 48 h negative control siRNA, BIRC5 siRNA (5 µM) and YM155 (5 nM) treatment as shown by Western blot. (B) Representative immunoflurosence staining of HIF-1α in 24 h 5 nM YM155-treated SACC-83 cell line as compared with vehicle treated counterpart in normoxia culture condition. Scale bars, 10 µm. (C) Quantification of HIF-1α nuclear translocation by Image J. Mean ± SEM, Student *t* test, **, *P*<0.01.

To further explore the regulation of survivin, HIF-1α, TGF-β1 and TFE3, human AdCC cell line SACC-83 were used to determine the role of survivin inhibition using BIRC5 siRNA as well small molecule YM155. As shown in Fig. 8A, 5 µM BIRC5 siRNA and 5 nM YM155 efficiently reduce survivin protein expression at 48 h treatment as indicated by Western blot (Fig. 8C). Interestingly, the expression of HIF-1α, TGF-β1 and TFE3 was also consistently reduced after suvivin inhibitoin (Fig. 8A). Immunofluorescence staining shows 5 nM YM155 treatment significant decrease nuclear expression of HIF-1α even at 24 h normoxic culture condition as shown in Fig. 8B and quantification in Fig. 8C (*P*<0.01).

## Discussion

Survivin, the most important member of the IAP family, has been implicated in both cell survival and cell cycle regulation in many cancers [36], [37]. In this research, the expression of survivin was consistent with data from previous studies [38], [39]. We reported not only the expression of survivin but also certain hypoxia related HIF-1α and TGF-β1 that were highly expressed in AdCC. Interestingly, we first demonstrated that the expression of TFE3, a novel transcription factor with the oncogenic potential of TGF-β1 [40], was significantly higher in AdCC as compared with NSG and PA. The similar expression patterns of HIF-1α, TGF-β1 and TFE3 indicate that the close correlation of survivin expression in AdCC may be correlated with hypoxia. To further address the question whether survivin regulates HIF-1α, TGF-β1 and TFE3, we investigated the role of survivin in regulating hypoxia related HIF-1α, TGF-β1 and TFE3 *in vitro* by using a specific survivin inhibitor (YM155) and BIRC5 siRNA. Interestingly, the results suggested that the inhibition of survivin by small molecule as well as siRNA can significantly downregulate the expression of HIF-1α, TGF-β1 and TFE3 *in vitro*. Therefore, HIF-1α, TGF-β1 and TFE3 may be downstream targets of survivin.

Hypoxia is believed to be one of the mechanisms in increasing tumor aggression and survival in AdCC [14]. TGF-β1 and HIF-1α has been proved to interdependent and interact in normoxic and hypoxic epithelial collagen expression [41]. By suppressing TGF-β1 signaling in the epithelia, the expression of survivin was elevated leading to the resistance to apoptosis in prostate epithelial cells [20]. Our previous data also indicated that the loss of function of TGF-β/TGFBR1 signaling in epithelia increased epithelial survivin expression and stromal TGF-β1 accumulation, with concomitant increase of HIF-1α translocation in the transgenic mouse model [42], [43]. The stromal TGF-β1 accumulation plays an important role in promoting angiogenesis and recruiting inflammatory cells that promote tumor progression [44]. This “chargeable battery” theory may partly explain how exogenous TGF-β1 can upregulate the expression of survivin and promote the growth of NPC TW01 cells [21]. However, there are no data to address the question of the regulation of TGF-β1 and HIF-1α by survivin. In this study, we not only confirm the close relationship between TGF-β1, HIF-1α and survivin in AdCC, but also confirm that the inhibition of survivin may decrease TGF-β1 and the expression of its downstream target protein, TFE3. By treating AdCC cell line with BIRC5 siRNA or YM155, a novel imidazolium-based compound[45], we observed a reduced cell proliferation, increase of apoptosis as well as autophagic cell death[30], which further demonstrates the tumor-promoting role of survivin in AdCC. Further analysis revealed that survivin inhibition may downregulate the protein expression of HIF-1α, TGF-β1 and TFE3 in AdCC. Taken together, the above data results suggest that HIF-1α, TGF-β1 and TFE3 play a tumor-promoting role and show a positive correlation with survivin in AdCC.

Our data suggest that the expression of survivin, HIF-1α, TGF-β1 and TFE3 is not only important to the tumorigenesis of AdCC but also has a diagnostic implication. All these markers are highly expressed in AdCC as compared with NSG, the benign salivary gland tumor (PA) and the salivary gland malignancy (MEC). To be honest, this study still has limitations because the cases of benign tumor and other salivary gland malignancies are not large in number, and it is hard to cover all the features of the specimen and histological type. In addition, this study has a rather large number of AdCC samples, and reveals potential diagnostic roles of survivin, HIF-1α, TGF-β1 and TFE3 in AdCC. We further investigate the role of these markers in the differential diagnosis of the pathological type of AdCC because several studies have identified pathological factors in AdCC with an unfavorable effect on survival, and a solid histological subtype may represent poor survival and high recurrence in AdCC [46]. The present data show a slight increase in the expression of survivin, HIF-1α and TFE3 in solid AdCC, though this increase did not reach statistical significance. Of interest, our data suggest survivin and HIF-1α significant increase in MEC, which indicate hypoxia may play similar important role of MEC. More importantly, TGF-β1 and TFE3 significantly increase in AdCC but not in MEC suggest the potential role of TGF-β1/TFE3 axis in AdCC progression, which may related to AdCC's unique biological behavior on EMT[47], distant metastasis and angiogenesis[48] as compared with MEC. Equal importantly, high expression of TGF-β1/TFE3 may have significance in differential diagnosis of AdCC from MEC.

Although the efficacy of radiotherapy in AdCC is lower as compared with other solid tumors, it is still one of the most commonly used treatments for AdCC [49]. One of the most important reasons for radioresistance is hypoxia [50]. Although the mechanism of radioresistance behind the hypoxia effect has not yet been fully elucidated, it is clear that hypoxia plays an important role in tumor recurrence after radiation therapy, and in poor prognosis of cancer patients after radiation therapy [51]. HIF-1α is important hypoxia-related factor that is significantly increased after radiotherapy and are closely related with the self-renewal of radioresistant cancer stem cells [11]. Please keep in mind that our data are in the primary stage. The exciting result that YM155 can reduce both HIF-1α and TGF-β1 in AdCC, suggests that survivin inhibition may attenuate radiotherapy induced hypoxia. The combined application of YM155 may have a radiosensitive effect. More importantly, survivin inhibition may target radiotherapy-induced cancer stem cells in AdCC or other solid tumors.

In conclusion, our findings suggest that survivin, HIF-1α, TGF-β1 and TFE3 have a close correlation and may play important roles in the tumorigenesis and progression of AdCC. The increased expression of TFE3 may be related to hypoxia. Targeting survivin by YM155 can reduce the HIF-1α, TGF-β1 and TFE3 of AdCC. Targeting survivin may be an effective and novel rationale to suppress the growth, improve the prognosis of AdCC and the potential radiosensitive effect by target cancer stem cells.

## Supporting Information

S1 Datasheet Digital quantification of immunohistochemical stainning of Survivin, HIF-1α, TGF-β1 and TFE3 in AdCC tissue microarray.
(XLS)
